# Capsule Networks Showed Excellent Performance in the Classification of hERG Blockers/Nonblockers

**DOI:** 10.3389/fphar.2019.01631

**Published:** 2020-01-28

**Authors:** Yiwei Wang, Lei Huang, Siwen Jiang, Yifei Wang, Jun Zou, Hongguang Fu, Shengyong Yang

**Affiliations:** ^1^ State Key Laboratory of Biotherapy and Cancer Center, West China Hospital, Sichuan University, Chengdu, China; ^2^ College of Preclinical Medicine, Southwest Medical University, Luzhou, China; ^3^ School of Computer Science and Engineering, University of Electronic Science and Technology of China, Chengdu, China; ^4^ Basic Teaching Department, Sichuan College of Architectural Technology, Deyang, China

**Keywords:** deep learning, hERG, classification model, Capsule network, convolution-capsule network, restricted Boltzmann machine-capsule networks

## Abstract

Capsule networks (CapsNets), a new class of deep neural network architectures proposed recently by Hinton et al., have shown a great performance in many fields, particularly in image recognition and natural language processing. However, CapsNets have not yet been applied to drug discovery-related studies. As the first attempt, we in this investigation adopted CapsNets to develop classification models of hERG blockers/nonblockers; drugs with hERG blockade activity are thought to have a potential risk of cardiotoxicity. Two capsule network architectures were established: convolution-capsule network (Conv-CapsNet) and restricted Boltzmann machine-capsule networks (RBM-CapsNet), in which convolution and a restricted Boltzmann machine (RBM) were used as feature extractors, respectively. Two prediction models of hERG blockers/nonblockers were then developed by Conv-CapsNet and RBM-CapsNet with the Doddareddy's training set composed of 2,389 compounds. The established models showed excellent performance in an independent test set comprising 255 compounds, with prediction accuracies of 91.8 and 92.2% for Conv-CapsNet and RBM-CapsNet models, respectively. Various comparisons were also made between our models and those developed by other machine learning methods including deep belief network (DBN), convolutional neural network (CNN), multilayer perceptron (MLP), support vector machine (SVM), k-nearest neighbors (kNN), logistic regression (LR), and LightGBM, and with different training sets. All the results showed that the models by Conv-CapsNet and RBM-CapsNet are among the best classification models. Overall, the excellent performance of capsule networks achieved in this investigation highlights their potential in drug discovery-related studies.

## Introduction

The human ether-a-go-go-related gene (hERG) encodes a potassium channel protein, which is important for cardiac electrical activity and the coordination of heartbeat. Blockade of the hERG potassium channel can result in a potentially fatal disorder called long QT syndrome, as well as serious cardiotoxicity, which has led to the withdrawal of several marketed drugs and the failure of many drug research and development projects (Fermini and Fossa, 2003; Recanatini et al., 2005; Sanguinetti and Tristani-Firouzi, 2006; Bowes et al., 2012; Nachimuthu et al., 2012; Zhang et al., 2012; Shah, 2013; Kalyaanamoorthy and Barakat, 2018; Mladenka et al., 2018). Therefore, drug candidates that can bind with hERG should be eliminated as early as possible in drug discovery studies. At present, various *in vitro* experimental assays, such as fluorescent measurements (Dorn et al., 2005), radioligand binding assay (Yu et al., 2014), and patch-clamp electrophysiology (Stoelzle et al., 2011; Gillie et al., 2013; Danker and Moller, 2014), have been developed to measure the hERG binding affinity of chemicals. Nevertheless, these assays are often expensive and time-consuming, implying that they are not suitable for the evaluation of hERG binding affinity for a large number of chemicals in the early stage of drug discovery. Furthermore, the preconditions for the use of these analytical techniques are that the chemical compounds have been synthesized and are available in hand, which are usually not applicable in the era of virtual high-throughput screening. An alternative strategy is to use *in silico* methods; compared with experimental assays, *in silico* methods are cheaper and faster, and also do not involve any of the aforementioned preconditions.

To date, various *in silico* prediction models have been developed for hERG channel blockade. These models can be classified into structure-based and ligand-based models. Structure-based models utilize molecular docking to predict the binding mode and binding affinity of compounds to hERG. However, the structure-based methods often have some limitations such as protein flexibility, inaccurate scoring function, and solvent effect (Jia et al., 2008; Li et al., 2013). Ligand-based models can further be classified into several categories based on structural and functional features (Zolotoy et al., 2003; Aronov, 2005), quantitative structure-activity relationship (QSAR) models (Perry et al., 2006; Yoshida and Niwa, 2006; Tan et al., 2012), pharmacophore models (Cavalli et al., 2002; Aronov, 2006; Durdagi et al., 2011; Yamakawa et al., 2012; Kratz et al., 2014; Wang et al., 2016), and machine learning models (Wang et al., 2008; Klon, 2010; Wacker and Noskov, 2018). Compared with other models, machine learning models have attracted more attention in recent years due to the remarkable performance of machine learning methods in the handling of classification issues. For example, Wang et al. (2012) established binary classification models using Naïve Bayes (NB) classification and recursive partitioning (RP) methods, which achieved prediction accuracies of 85–89% in their test sets. Zhang and coworkers (Zhang et al., 2016) used five machine learning methods to develop models that can discriminate hERG blockers from nonblockers, and they found that k-nearest neighbors (kNN) and support vector machine (SVM) methods showed a better performance than others. Broccatelli et al. (2012) derived several classification models of hERG blocker/nonblocker by using random forests (RF), SVM, and kNN algorithms with descriptor selections *via* genetic algorithm (GA) methods, and their prediction accuracies ranged from 83 to 86%. Didziapetris and Lanevskij (2016) employed a gradient-boosting machine (GBM) statistical technique to classify hERG blockers/nonblockers, and this offered overall prediction accuracies of 72–78% against different test sets. Very recently, Siramshetty et al. (2018) employed three methods (kNN, RF, and SVM) with different molecular descriptors, activity thresholds, and training set compositions to develop predictive models of hERG blockers/nonblockers, and their models showed better performance than previously reported ones.

There have been remarkable advances in deep learning methods since a fast learning algorithm for deep belief nets was proposed by Hinton in 2006 (Hinton et al., 2006a). They have widely been applied to fields particularly computer vision, speech recognition, natural language processing, audio recognition, social network filtering, machine translation, bioinformatics, and various games (Collobert and Weston, 2008; Bengio, 2009; Dahl et al., 2012; Hinton et al., 2012; LeCun et al., 2015; Defferrard et al., 2016; Mamoshina et al., 2016), where they have produced results comparable to or in some cases superior to human experts. In recent years, deep learning has also been applied to drug discovery, and it has demonstrated its potentials (Lusci et al., 2013; Ma et al., 2015; Xu et al., 2015; Aliper et al., 2016; Mayr et al., 2016; Pereira et al., 2016; Subramanian et al., 2016; Kadurin et al., 2017; Ragoza et al., 2017; Ramsundar et al., 2017; Xu et al., 2017; Ghasemi et al., 2018; Harel and Radinsky, 2018; Hu et al., 2018; Popova et al., 2018; Preuer et al., 2018; Russo et al., 2018; Segler et al., 2018; Shin et al., 2018; Cai et al., 2019; Wang et al., 2019a; Yang et al., 2019). However, there are still some issues that limit the application of deep learning in drug discovery. For example, deep learning usually requires a large number of samples for model training. Unfortunately, there are often a very limited number of agents (usually hundreds or thousands) in drug discovery-related studies due to high cost and the lengthy process involved in obtaining samples and their associated properties. In addition, commonly used deep learning algorithms or networks, such as convolutional neural network (CNN), are primarily designed for two-dimensional (2D) image recognition. In these networks, some special algorithms, such as the pooling algorithm in CNN, are adopted to reduce the dimensionality of the representation, which might lead to a loss of information.

To overcome the shortcomings of traditional deep learning networks, Hinton group (Sabour et al., 2017) proposed new deep learning architectures known as capsule networks (CapsNets), which introduced a novel building block that is used in deep learning to improve the model hierarchical relationships inside the internal knowledge representation of a neural network. CapsNets have shown great potential in some fields (Xi et al., 2017; Afshar et al., 2018; Lalonde and Bagci, 2018; Qiao et al., 2018; Vesperini et al., 2018; Zhao et al., 2018; Wang et al., 2019b; Peng et al., 2019). However, CapsNets have not yet been applied to drug discovery-related studies. As the first attempt, in this study, we established two classification models of hERG blockers/nonblockers by using modified capsule network architectures. The models were evaluated using a test set and an external validation set, which are independent of the training set. Furthermore, our models were also compared with others.

The rest of this paper is organized as follows. The *Materials and Methods* section describes the implementation of the two capsule networks [convolution-capsule network (Conv-CapsNet) and RBM-CapsNet] developed in this study, as well as the data sets used and computational modeling details. The modeling, evaluation, and comparison with other models are presented in the *Results* section. The strengths of the capsule networks are analyzed in the *Discussion* section which is followed by a final summary.

## Materials and Methods

### Convolution-Capsule Network

#### Architecture

**Figure 1:**
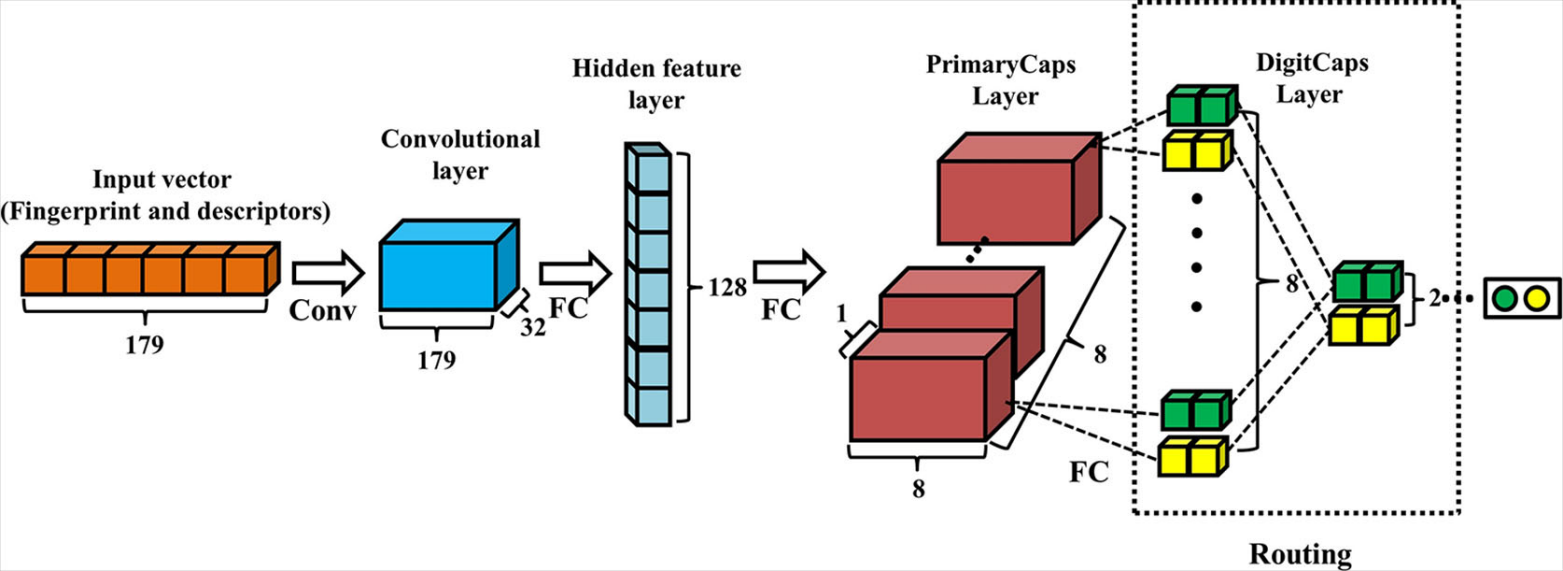
Architecture of convolution-capsule networks (Conv-CapsNet). The input is one-dimensional vector containing 179 components. The convolution layer has 32 filters of size 1×3. The hidden feature layer and PrimaryCaps layer consist of 128 and 64 nodes, respectively. The weight matrix between PrimaryCaps layer and DigitCaps layer is 8×8×2×2, and two dynamic routing iterations were adopted.

The architecture of Conv-CapsNet is schematically shown in **Figure 1**, which is similar in nature to that of the Hinton's original Capsule Network, except for one additional hidden feature layer. Apparently, Conv-CapsNet contains four layers: a convolutional layer, a hidden feature layer, a PrimaryCaps layer, and a DigitCaps layer. It is composed of 179 nodes for input, which are based on the feature vector size of the molecules. With mapping from the input vector, the hidden feature layer with 128 dimensional nodes was generated by one convolutional operation and one fully connected operation. The PrimaryCaps layer comprises eight capsules (*u_i_*), and each capsule in this layer includes eight-dimensional features. Furthermore, we computed the contribution (${\hat{u}}_{j|i}$) of each capsule (*u_i_*) in PrimaryCaps to that (*v_j_*) in DigitCaps by using Eq. 1.

(1) $${\hat{u}}_{j|i}=W_{ij}\cdot u_{i}$$

The final layer (DigitCaps) has a two-dimensional capsule ($v_{j}$) per digit class (two classes in this investigation). Each of these capsules received input from all the capsules in the PrimaryCaps layer through Eq. 2-1, Eq. 2-2, and Eq. 2-3.

(2-1) $$c_{ij}=\frac{\exp\left(b_{ij}\right)}{\Sigma_{k}\exp\left(b_{ik}\right)}$$

(2-2) $$s_{j}=\Sigma_{k}c_{ij}{\hat{u}}_{j|i}$$

(2-3) $$v_{j}=\frac{{\left\|s_{j}\right\|}^{2}}{1+{\left\|s_{j}\right\|}^{2}}\frac{s_{j}}{\left\|s_{j}\right\|}$$

Finally, we computed the length of each digit capsule to predict the class of chemical molecules from Eq 3.

(3) $$L_{k}=T_{k}\max{\left(0,m^{+}-\left\|v_{k}\right\|\right)}^{2}+\lambda \left(1-T_{k}\right)\;\max{\left(0,\left\|v_{k}\right\|-m^{-}\right)}^{2}$$

In view of the small size of the dataset in this account, we added the L2 regularization behind the convolutional operation to prevent the network from overfitting (Ng, 2004).

#### Hyperparameter Optimization

For the hyperparameter optimization of the Conv-CapsNet architecture, the different numbers of filters in the convolutional layer, nodes in the hidden feature layer, and dimensions in PrimaryCaps were explored. Additionally, the dynamic routing iterations between two capsule layers were tested from 1 to 3 with an increment of 1. For each group of the parameter settings, the performance of the model was evaluated by five-fold cross-validation based on the training set. Once the highest accuracy was achieved with all the candidate settings, the best setting was subsequently applied to the test set and external validation set. We employed early stopping to reduce the overfitting problem, which is a technique commonly used for the reduction of overfitting (Caruana et al., 2001). With the early stopping, original training set was randomly divided into a new training set and a validation set (4:1). When the error in the validation set was less than that from the previous iteration, the training was immediately stopped. The final optimal hyperparameters for Conv-CapsNet are listed in **Table 1**.

**Table 1:** Hyperparameter settings of convolution-capsule networks (Conv-CapsNet).

Hyperparameter	Setting
L2 normalization term	0.001
Activation	Relu
Batch size	148
Iteration epoch	300
Learning rate of network	0.001
Optimizer	Adam
Filter	32
Kernel_size	3
Number of nodes in the hidden feature layer	128
Number of nodes in the PrimaryCaps layer	64
Routing time	2
Dimension of each capsule	8
Length of PrimaryCaps	2
Length of DigitCaps	2

#### Model Training of Conv-CapsNet

The Conv-CapsNet weights were randomly initialized using a truncated normal distribution with the standard deviation being set as 0.01 during training. Both the convolutional and hidden feature layers adopted the rectified linear unit (Relu) as the activation function. To reduce the internal-covariate-shift, we used batch normalization to normalize the input distribution of each layer to a standard Gaussian distribution (Hinton et al., 2011; Ioffe and Szegedy, 2015). The adaptive moment estimation (Adam) method was employed for optimization (Kingma and Ba, 2014).

**Table 2** summarizes the algorithm and training procedure for Conv-CapsNet. *CW*, *W*1, and *W*2 represent the parameters in the convolutional, hidden feature, and PrimaryCaps layers, respectively. The convolutional and the first two fully connected operations are represented by *conv*, *fc*1, and *fc*2, respectively; *conv_layer, hf_layer*, and *pc_layer* denote the output from the convolutional, hidden feature, and PrimaryCaps layers, respectively. Through a feature vector extraction process in the convolutional layer, the hidden feature layer, and the PrimaryCaps layer (lines 1–4), *pc_layer* was packed as capsules *u* (line 5). Here, $\hat{u}$ denotes the contribution of one layer to the next layer. Next, the routing algorithm was used to generate the digit capsules (lines 6–13). Len is the length of the output of DigitCaps layer (lines 14). Lines 15–20 are for the network parameter update using a gradient step.

**Table 2:** Algorithm and training procedure of convolution-capsule networks (Conv-CapsNet).

**Algorithm:** Conv-CapsNet training algorithm, using a mini-batch stochastic gradient descent (SGD) for simplicity.
**Input:** mini batch feature vector **(*x*)**; Number of Conv-CapsNet training epoch **(S)**; Number of dynamic routing iterations **(iter)**. **Output:** Length of each capsules **(Len)**.
1: **For** n=1 **to** S **do** 2: *conv_layer* ← *conv*(*x*, *CW*) 3: *hf_layer* ← *fc*1(*conv_layer*, *W*1) 4: *pc_layer* ← *fc*2(*hf_layer*, *W*2) 5: *u* ← Encapule(*pc_layer*) 6: For all capsule *i* in PrimaryCaps layer:${\hat{u}}_{j|i}\leftarrow W_{ij}u_{i}$………… {contribution computes Eq. 1} 7: For all capsule *i* in PrimaryCaps layer and capsule *j* in DigitCaps layer:*b_ij_* ← 0 8: **For** *m=1 to* **iter do** 9: For all capsule *i* in PrimaryCaps layer: *c_i_* ← *softmax*(*b_i_*) ……{softmax computes Eq. 2-1} 10: For all capsule *j* in DigitCaps layer:$s_{j}\leftarrow \sum_{i}c_{ij}{\hat{u}}_{j|i}$ …{dynamic computes Eq. 2-2} 11: For all capsule *j* in DigitCaps layer: *v_j_* ← *squash* (*s_j_*) ………{squash computes Eq. 2-3} 12: For all capsule *i* in PrimaryCaps layer and capsule *j* in DigitCaps layer:$\;b_{ij}\leftarrow b_{ij}+{\hat{u}}_{j|i}\cdot v_{j}$ 13: **End for** 14: Len←Length of v
15: L←loss of v………………………………………{loss computes Eq. 3} 16: W←W−∂L/∂W 17: CW←CW−∂L/∂CW 18: W1←W1−∂L/∂W1 19: W2←W2−∂L/∂W2 20: **End for**

### Restricted Boltzmann Machine-Capsule Network

#### Architecture

**Figure 2:**
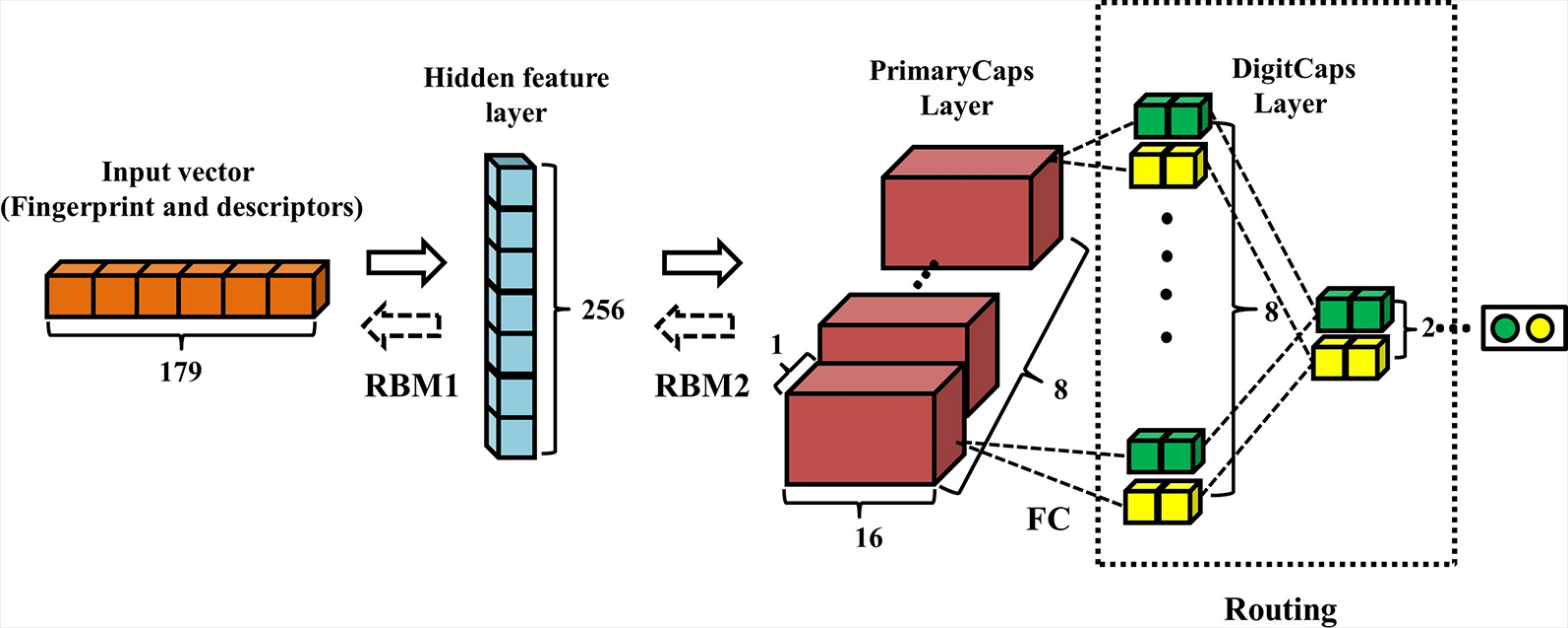
Architecture of restricted Boltzmann machine-capsule networks (RBM-CapsNet). The input is one-dimensional vector containing 179 components. The hidden feature layer and PrimaryCaps layer consist of 256 and 128 nodes, respectively. The weight matrix between PrimaryCaps layer and DigitCaps layer is 8×8×2×2, and two dynamic routing iterations were adopted.

**Figure 2** displays the architecture of RBM-CapsNet, which consists of three layers: a hidden feature layer, a PrimaryCaps layer, and a DigitCaps layer. In RBM-CapsNet, two restricted Boltzmann machines (RBMs) replaced the convolutional and fully connected operations in Conv-CapsNet. The first RBM encodes the original vector (179-dimension) for the feature space (the hidden feature layer), which is subsequently used as the input for the next RBM. The RBMs used energy function (Eq. 4) as the loss function (Hinton and Salakhutdinov, 2006b).

(4) $$E\left(v,h\right)=-\left(a^{T}\cdot v+b^{T}\cdot h+v^{T}\cdot \omega \cdot h\right)$$

The capsule networks still consist of PrimaryCaps and DigitCaps, which are the same as in Conv-CapsNet. The detailed definitions of all the parameters in Eq. 1, 2, 3, and 4 are listed in the **Supplementary Material**.

#### Hyperparameter Optimization

To optimize the hyperparameters in the RBM-CapsNet architecture, all the combinations of one to five RBM operations and 32, 64, 128, 256, and 512 nodes in each RBM were tested. The basic optimization procedure for the hyperparameters related to the capsules is very similar with that for Conv-CapsNet. The performance of each RBM-CapsNet architecture was examined by five-fold cross-validation. The candidate RBM-CapsNet architecture that provided the highest accuracy was validated using the test set and external validation set. The detailed information on the optimized hyperparameters in RBM-CapsNet is summarized in **Table 3**.

**Table 3:** Hyperparameter settings of restricted Boltzmann machine-capsule networks (RBM-CapsNet).

Hyperparameter	Setting
Numbers of RBM	2
Number of nodes in the hidden feature layer	256
Number of nodes in the PrimaryCaps layer	128
Iteration of RBM	100
Iteration of network	200
Learning rate of RBM	0.001
Learning rate of network	0.005
Activation	Relu
Batch size	148
Optimizer	Adam
Routing time	2
Dimension of each capsule	8
Length of PrimaryCaps	2
Length of DigitCaps	2

#### Model Training of Restricted Boltzmann Machine-Capsule Network

The training process was divided into two stages. First, two RBMs were pre-trained one by one with the loss function shown in Eq. 4. Second, the parameters of RBMs from pre-training were taken as initial values and the whole network was fine-tuned by back-propagation algorithm with end-to-end (Rumelhart et al., 1986).

**Table 4** summarizes the algorithm and training procedure for RBM-CapsNet. *θ*1 and *θ*2 represent the parameters of the hidden feature and PrimaryCaps layers, respectively. *ϕ*1 and *ϕ*2 represent the operations in RBM1 and RBM2, respectively. The *hf_layer* and *pc_layer* denote the output from the hidden feature and PrimaryCaps layers, respectively. After training RBM1 and RBM2 individually (lines 1–6), the *pc_layer* was packed as capsules *u* (line 10). The routing algorithm was then used to generate the digit capsules (lines 11–18). Len is the length of the output of DigitCaps layer (lines 19). Lines 20 to 24 are for a network parameter update using a gradient step (∂*L/*∂*W* represents the gradient of the contribution matrix, and ∂*L/*∂*θ*1 and ∂*L/*∂*θ*2 represent the gradients of the parameters for the hidden feature and PrimaryCaps layers, respectively).

**Table 4:** Algorithm and training procedure of restricted Boltzmann machine-capsule networks (RBM-CapsNet).

**Algorithm:** RBM-CapsNet training algorithm, using a mini-batch stochastic gradient descent (SGD) for simplicity.
**Input:** mini batch feature vector **(*x*)**; Number of RBM training epoch **(S1)**; Number of Capsule training epoch **(S2)**; Number of dynamic routing iterations **(iter)**. **Output:** Length of each capsules **(Len).**
1: **For** n=1 **to** S1 **do** 2: hf_layer←ϕ1(x,θ1)………………………………………{RBM1 training} 3: **End for** 4: **For** n=1 **to** S1 **do** 5: pc_layer←ϕ2(hf_layer,θ2)…………………………………{RBM2 training} 6: **End for**
7: **For** n=1 **to** S2 **do** 8: hf_layer←ϕ1(x,θ1) 9: pc_layer←ϕ2(h1_layer,θ2) 10: u←Encapule (pc_layer ) 11: For all capsule *i* in PrimaryCaps layer:${\hat{u}}_{j|i}\leftarrow W_{ij}u_{i}$…………{contribution computes Eq. 1} 12: For all capsule *i* in PrimaryCaps layer and capsule *j* in DigitCaps layer:$b_{ij}\leftarrow 0$ 13: **For** *m=1 to* **iter do** 14: For all capsule *i* in PrimaryCaps layer: ……{softmax computes Eq. 2-1} 15: For all capsule *j* in DigitCaps layer:$\;s_{j}\leftarrow \;\sum_{i}c_{ij}{\hat{u}}_{j|i}$………{dynamic computes Eq. 2-2} 16: For all capsule *j* in DigitCaps layer:$v_{j}\leftarrow squash\left(s_{j}\right)$…………{squash computes Eq. 2-3} 17: For all capsule *i* in PrimaryCaps layer and capsule *j* in DigitCaps layer: $\;b_{ij}\leftarrow b_{ij}+{\hat{u}}_{j|i}\cdot v_{j}$ 18: **End for**
19: Len←Length of v 20: L←loss of v………………………………………{loss computes Eq. 3} 21: W←W−∂L/∂W 22: θ1←θ1−∂L/∂θ1 23: θ2←θ2−∂L/∂θ2 24: **End for**

### Data Sets

In this investigation, the Doddareddy's hERG blockade data set was used to establish our models (Doddareddy et al., 2010), which includes literature compounds tested on the hERG channel and Food and Drug Administration (FDA)-approved drugs. This data set contains a total of 2,644 compounds, including 1,112 positives (hERG blocker, IC_50_ < 10 μM) and 1,532 negatives (hERG nonblocker, IC_50_ > 30 μM). Doddareddy et al. partitioned this data set into a training set and a test set (Doddareddy et al., 2010). For comparison, the same partition scheme for the training and test sets as that by Doddareddy et al. was adopted in this investigation. Furthermore, we used Doddareddy's experimentally validated dataset (a total of 60 compounds: 50 agents from the Chembridge database and 10 from an in-house compound library) as an external validation set to assess the generalization ability of our models. In order to compare the performance of our models with others reported in the literature, we also used the same data sets as those in the literature, including Hou's (Wang et al., 2012; Wang et al., 2016), Zhang's (Zhang et al., 2016), Sun's (Sun et al., 2017), Siramshetty's (Siramshetty et al., 2018), and Cai's (Cai et al., 2019) data sets. Here, it is necessary to mention that an integrated data set of hERG blockade, which is the largest database to date, has been collected by Sato et al. (2018). However, we did not use this data set because it was not accessible. Another reason was that this data set has not been used to develop prediction models so far, and hence, a comparison study involving the data set was not feasible.

### Molecular Characterization

In this investigation, a combination of MACCS (MDL Molecular Access) molecular fingerprints (166 bits) and 13 molecular descriptors was utilized to characterize the chemical compounds, which has been used by Zhang et al. and showed a good predictive performance in hERG blockade classification modeling (Zhang et al., 2016). Another reason why we adopted this characterization method (MACCS+13 descriptors, a total of 179 features) is because of their short length which is important for the reduction of the number of parameters in the modeling and the training time. By the way, the 13 molecular descriptors were selected because they are thought to be very related to ADMET properties and have been widely used in the modeling of various ADMET properties (Hou and Wang, 2008; Hou et al., 2009; Wang et al., 2012; Zhang et al., 2016). A detailed list of these descriptors are given as follows: the octanol-water partitioning coefficient, apparent partition coefficient at pH = 7.4, molecular solubility, molecular weight, number of hydrogen bond donors, number of hydrogen bond acceptors, number of rotatable bonds, number of rings, number of aromatic rings, sum of the oxygen and nitrogen atoms, polar surface area, molecular fractional polar surface area, and molecular surface area.

All the molecular fingerprints and molecular descriptors were computed with RDKit (Landrum, 2018) and PaDEL-Descriptor (Yap, 2011), respectively. Because the values of the different descriptors might span significantly different numerical ranges, their values were scaled to the same range (0, 1) by using the following formula:

(5) $$x^{*}=\frac{x-\min}{\max-\min}$$

where *x* is the original value, x* is the scaled value, and max and min are the maximum and minimum values of a descriptor, respectively.

### Model Assessment

All the models were assessed based on their accuracy (Q), sensitivity (SE), and specificity (SP). Q reflects the total prediction effect of a classifier. SE and SP represent the predictive power for positives and negatives, respectively. The definitions are given as follows (TP, true positive/blocker; TN, true negative/nonblocker; FP, false positive/blocker; and FN, false negative/nonblocker):

(6) $$Q=\frac{TP+TN}{TP+FP+TN+FN}$$

(7) $$SE=\frac{TP}{TP+FN}$$

(8) $$SP=\frac{TN}{FP+TN}$$

The classification capability of models was measured by area under the receive operating characteristic curve (AUC), which is an important indicator to illustrate the classification performance by changing its discrimination threshold.

Another measurement of the quality of binary (two-class) classifications is the Matthew's correlation coefficient (MCC). The MCC considers the balance ratios of the four confusion matrix categories (TP, TN, FP, and FN), and reflects the predictive power of models objectively without the influence of the disproportionate ratio of positives to negatives in the dataset. The MCC was calculated by using the following equation:

(9) $$MCC=\frac{TP\times TN-FP\times FN}{\sqrt{\left(FP+TN\right)\left(FP+TP\right)\left(FN+TN\right)\left(FN+TP\right)}}$$

### Computations

All the calculations were carried out with a single dual-processor, 16-core 2.1 GHz Intel® Xeon® E5-2683 v4 CPU with 126 GB memory and two NVIDIA Tesla P100 GPU accelerators. The software modules that were used to implement this project included Scikit-learn 0.18.1, Python 3.6.4, Anaconda 5.1.0 (64-bit), and TensorFlow 1.4.0.

## Results

### Selection of the Optimal Capsule Network Architectures and Model Development

Hinton et al raised the concept of capsule network and proposed the first capsule network architecture prototype (Sabour et al., 2017). To find the optimal capsule network architectures for the modeling of hERG blockade, we tried to construct a number of capsule networks with different architectures following Hinton's principle. Here, the Doddareddy's training set (positives: 1,004; negatives: 1,385) was adopted to train all the models, and the five-fold cross-validation method was used to monitor the training processes. In the five-fold cross-validation, the training set was randomly divided into five subsets. Of the five subsets, four subsets were used as the training data, and the remaining subset was used as the validation data for testing the model. The cross-validation process was repeated five times, with each of the five subsets used exactly once as the validation data. The average of the results from the five runs was calculated to produce a single estimation. The five-fold cross-validation results for the training set are given in **Table 5**. According to these results, Conv-CapsNet and RBM-CapsNet showed the best performance. For the Conv-CapsNet model, the prediction accuracies for the hERG blockers (SE) and the hERG nonblockers (SP) were 88.6 and 89.1%, respectively, and the overall prediction accuracy (Q) was 88.9%. For the RBM-CapsNet model, the prediction accuracies for hERG blockers and nonblockers were 84.3 and 89%, respectively and the overall prediction accuracy was 87.0%. Importantly, the MCC values of Conv-CapsNet and RBM-CapsNet were 0.774 and 0.734, respectively, which were also the highest among all the MCC values (**Table 5**); a higher MCC value often indicates a better prediction power of model. Therefore, the architectures of Conv-CapsNet and RBM-CapsNet were chosen as our capsule network architectures, and a detailed description for these architectures was given in the *Materials and Methods* section.

**Table 5:** Prediction results of hERG blockers/nonblockers classification models developed by capsule networks with different architectures.

Capsule network architecture	SE	SP	MCC	SD	Q (%)
Original CapsNet	80.4%	86.7%	0.673	0.0141	84.1%
FC+FC	82.6%	86.7%	0.694	0.0195	85.0%
Conv+FC	82.2%	86.4%	0.687	0.0166	84.6%
Conv+FC+FC **(Conv-CapsNet)**	**88.6%**	**89.1%**	**0.774**	**0.0109**	**88.9%**
Conv+Conv+FC+FC	84.5%	85.3%	0.693	0.0142	84.9%
Conv+Conv+Conv+FC+FC	81.9%	86.9%	0.685	0.0173	84.9%
One RBM	83.1%	86.5%	0.694	0.0182	84.9%
Two RBMs **(RBM-CapsNet)**	**84.3%**	**89.0%**	**0.734**	**0.0160**	**87.0%**
Three RBMs	84.5%	85.5%	0.696	0.0160	85.0%
Four RBMs	81.2%	86.0%	0.673	0.0108	83.9%
Five RBMs	84.1%	86.4%	0.701	0.0156	85.4%

*Conv, convolutional operation; FC, fully connected operation; RBM, restricted Boltzmann machine; Conv-CapsNet, convolution-capsule network; RBM-CapsNet, restricted Boltzmann machine-capsule network (The training set used was the Doddareddy's training set, and five-fold cross-validation was used to monitor the training performance. SE (%), sensitivity; SP (%), specificity; MCC, Matthew's correlation coefficient; SD, standard deviation; Q (%), overall accuracy). Conv-CapsNet and Conv-CapsNet showed the best performance.

### Validation of Our Models’ Prediction Ability Against hERG Blockers/Nonblockers by Doddareddy’s Test Set and External Validation Set

In the above subsection, we obtained the optimal architectures of capsule networks. With these capsule network architectures, two classification models of hERG blockers/nonblockers, Conv-CapsNet and RBM-CapsNet models have been developed. To verify the predictive ability of these models, two test sets that are independent of the training set were used: Doddareddy's test set (positives: 108; negatives: 147) and external validation set (positives: 18; negatives: 42).

**Figure 3:**
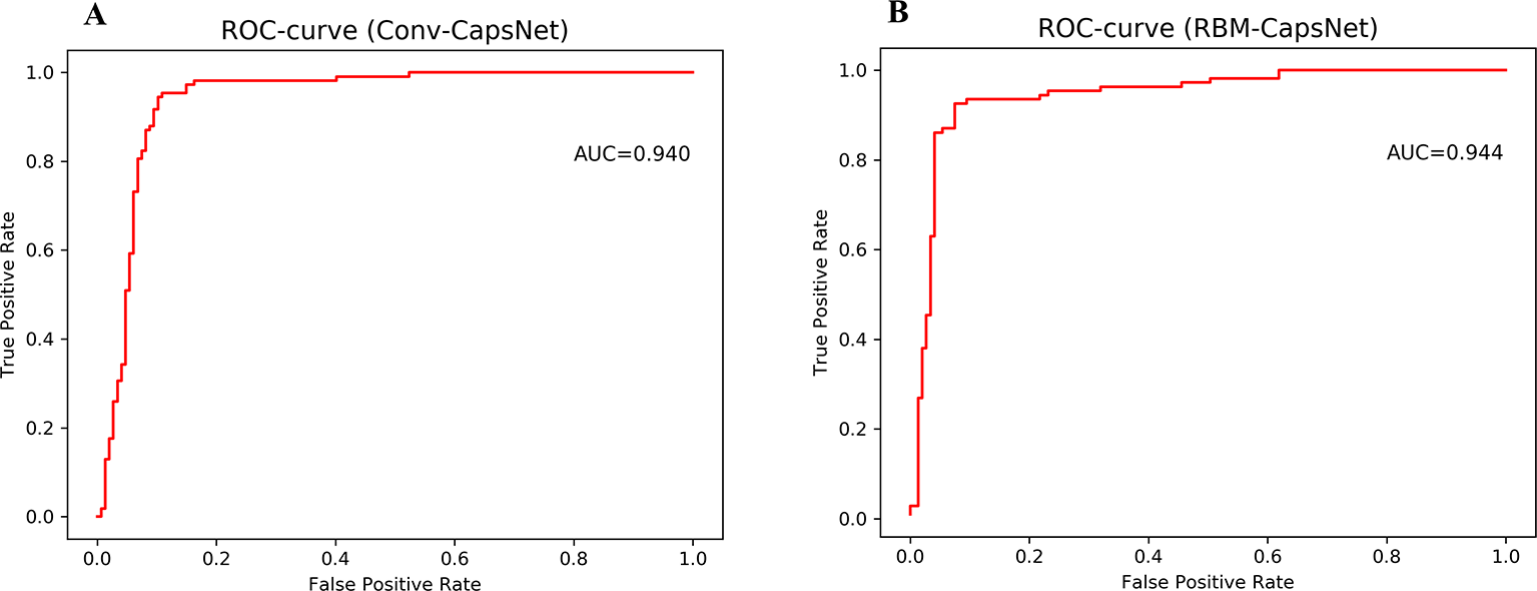
Receiver operating characteristic (ROC) curves for Doddareddy's test set by **(A)** convolution-capsule networks (Conv-CapsNet) and **(B)** restricted Boltzmann machine-capsule networks (RBM-CapsNet), respectively.

**Table 6** summarizes the prediction results of the Conv-CapsNet and RBM-CapsNet models. From **Table 6**, we can see that both models show excellent prediction ability to the Doddareddy's test set and external validation set. With the Conv-CapsNet model, of the 108 blockers in the test set, 102 were correctly predicted, indicating a prediction accuracy of 94.4% for the blockers (SE). For the 147 nonblockers, 132 (TN) were properly predicted. The accuracy for the prediction of nonblockers (SP) was 89.8%. Of all the 255 agents (blockers and nonblockers), 234 were correctly predicted and 20 were wrongly predicted (see **Table 6**). The overall prediction accuracy (Q) and AUC measure were 91.8% and 0.940 (see **Figure 3**), respectively. For the external validation set, of the 18 blockers, 16 (TP) were correctly discriminated from nonblockers. The prediction accuracy for the blockers (SE) was 88.9%. Of the 42 nonblockers, 30 (TN) were correctly predicted, indicating a prediction accuracy of 71.4% for the nonblockers (SP). Totally, 46 out of 60 compounds were correctly predicted. The overall prediction accuracy (Q) and AUC measure were 76.7% and 0.806, respectively. With the RBM-CapsNet model, in the test set, 99 (TP) out of 108 blockers were correctly predicted, indicating a prediction accuracy of 91.7%. Out of 147 nonblockers, 136 (TN) were correctly predicted, indicating a prediction accuracy of 92.5% for nonblockers. This model achieved an overall prediction accuracy of 92.2%. For the external validation set, the prediction accuracies for blockers (SE) and nonblockers (SP) were 94.4 and 71.4%, respectively. The overall prediction accuracy (Q) and the MCC values were 78.7% and 0.604, respectively. AUC for the test and external validation sets were 0.944 (see **Figure 3**) and 0.811, respectively. All of these results clearly demonstrate that the established Conv-CapsNet and RBM-CapsNet models can not only correctly classify the training set compounds but also have an outstanding predictability for external agents outside of the training set.

**Table 6:** Prediction accuracies of hERG blockade classification models developed by different methods with the same Doddareddy's training set.

Model	SE	SP	MCC	Q (%)	AUC
Doddareddy's test set (255/P:108, N:147)					
Conv-CapsNet	94.4%	89.8%	0.835	91.8%	0.940
RBM-CapsNet	91.7%	92.5%	0.840	92.2%	0.944
CNN	87.0%	85.0%	0.715	85.9%	0.933
MLP	82.4%	86.4%	0.687	84.7%	0.920
DBN	72.2%	80.8%	0.533	80.8%	0.903
SVM	90.7%	84.4%	0.743	87.1%	0.933
kNN	69.4%	96.6%	0.703	85.1%	0.928
Logistic regression	88.8%	83.7%	0.710	85.5%	0.858
LightGBM	79.6%	82.3%	0.617	81.2%	0.810
Doddareddy's external validation (60/P:18, N:42)					
Conv-CapsNet	88.9%	71.4%	0.554	76.7%	0.806
RBM-CapsNet	94.4%	71.4%	0.604	78.7%	0.811
CNN	94.4%	52.4%	0.441	65.0%	0.725
MLP	88.9%	57.1%	0.426	66.7%	0.707
DBN	88.9%	52.4%	0.386	63.3%	0.683
SVM	88.9%	52.4%	0.386	63.3%	0.660
kNN	77.8%	52.4%	0.279	60.0%	0.624
Logistic regression	83.3%	52.4%	0.332	61.7%	0.623
LightGBM	61.1%	59.5%	0.190	60.0%	0.609

(TP, true positive; TN, true negative; FP, false positive; FN, false negative; SE (%), sensitivity, SE = TP/(TP + FN); SP (%), specificity, SP = TN/(TN + FP); Q (%), overall accuracy, Q = [TP + TN)/(TP + TN + FP + FN)].

### Comparison of Our Models With Other Models Developed With the Same Doddareddy’s Training Set

To compare the performance of our models with that of others, we adopted commonly used machine learning methods to develop prediction models of hERG blockers/nonblockers with the same Doddareddy's training set. These machine learning methods include deep belief network (DBN), CNN, multilayer perceptron (MLP), SVM, kNN, logistic regression (LR), and LightGBM. Hyperparameters for these methods were optimized by five-fold cross-validation in advance, and the optimal hyperparameter values are listed in **Tables S1–S4**, respectively. The prediction results to the Doddareddy's test set and external validation set are also given in **Table 6**. From **Table 6**, we can see that the prediction accuracies of the seven models are obviously lower than those of our Conv-CapsNet and RBM-CapsNet models.

### Comparison of Our Models With Other Models Developed With Training Sets Different From Doddareddy’s Training Set

It has been well known that the performance of a prediction model is often strongly dependent on the training set used. Therefore, to make a more objective comparison, we collected various hERG blockade classification models developed with training sets different from Doddareddy's training set. With these training sets, we established a series of new prediction models by the Conv-CapsNet and RBM-CapsNet methods. To avoid a possible influence of molecular features, the same molecular features used in the literature were used. **Table 7** summarizes the prediction accuracies of various models reported in the literature together with those of models by Conv-CapsNet and RBM-CapsNet.

**Table 7:** Prediction results of various hERG blockade classification models developed with training sets different from Doddareddy's training set.

Entry	Model	Training set	Test set	SE	SP	Q
1	RP (Wang et al., 2016)	Hou's training set 1 (P: 283; N: 109)	Hou's test set 1 (P: 129; N: 66)	79.8%	75.8%	78.5%
	NB (Wang et al., 2016)			82.2%	75.8%	80.0%
	SVM (Wang et al., 2016)			90.7%	65.2%	82.1%
	Conv-CapsNet			85.7%	78.8%	82.0%
	RBM-CapsNet			84.1%	80.3%	82.0%
2	RP (Wang et al., 2016)	Hou's training set 2 (P: 272; N: 120)	Hou's test set 2 (P: 140; N: 55)	80.0%	74.5%	78.5%
	NB (Wang et al., 2016)			81.4%	80.0%	81.0%
	SVM (Wang et al., 2016)			85.0%	74.5%	82.1%
	Conv-CapsNet			82.1%	81.8%	82.0%
	RBM-CapsNet			81.4%	83.6%	82.0%
3	Bayesian (Wang et al., 2012)	Hou's training set 3 (P: 314; N: 306)	Hou's test set 3 (P: 63; N: 57)	86.9%	83.1%	85.0%
	Conv-CapsNet			87.3%	86.0%	86.8%
	RBM-CapsNet			88.9%	84.2%	86.8%
4	SVM (Zhang et al., 2016)	Zhang's training set (P: 717; N: 210)	Zhang's test set (P: 188; N: 48)	95.8%	34.0%	83.5%
	kNN (Zhang et al., 2016)			92.6%	40.4%	82.2%
	Conv-CapsNet			88.8%	66.7%	84.5%
	RBM-CapsNet			90.4%	64.6%	85.2%
5	LibSVM (Siramshetty et al., 2018)	Sun's training set (P: 483; N: 2541)	Sun's test set (P: 53; N: 13)	68.0%	85.0%	71.0%
	RF (Siramshetty et al., 2018)			72.0%	85.0%	74.0%
	Conv-CapsNet			83.0%	84.6%	83.3%
	RBM-CapsNet			86.8%	84.6%	86.3%
6	LibSVM (Siramshetty et al., 2018)	Siramshetty's training set T3 (P: 1406; N: 1708)	Doddareddy's test set (P: 108; N: 147)	64.0%	89.0%	78.0%
	RF (Siramshetty et al., 2018)			68.0%	91.0%	81.0%
	Conv-CapsNet			85.2%	88.4%	87.1%
	RBM-CapsNet			83.3%	91.2%	87.8%

Entry 1–3 of **Table 7** list models developed with Hou's training set 1 (positives: 283; negatives: 109), training set 2 (positives: 272; negatives: 120), and training set 3 (positive: 314; negative: 306), respectively. In Hou's training sets 1 and 2, a threshold of 40 µM was used to distinguish hERG blockers and nonblockers (blockers: IC_50_ < 40 µM; nonblockers: IC_50_ ≥ 40 µM). With training sets 1 and 2, Hou et al. established three models by RP, NB, and SVM methods, and the SVM models showed the best performance on their test sets. In Hou's training set 3, a threshold of 30 µM was used to define hERG blockers and nonblockers. A Bayesian classification model developed by Hou et al. with Hou's training set 3 gave a prediction accuracy of 85% on their test set. With Hou's training sets 1–3, we also separately established models by Conv-CapsNet and RBM-CapsNet methods. As shown in **Table 7**, our models showed comparable or superior performance compared with Hou's models. Entry 4 in **Table 7** shows models established by Zhang's training set (positives: 717; negatives: 210), in which a threshold of 30 µM was used to define hERG blockers and nonblockers. With the training set, Zhang et al. built two models by using SVM and kNN methods, which gave prediction accuracies of 83.5 and 82.2%, respectively, on their test set. Our models, developed by Conv-CapsNet and RBM-CapsNet, exhibited a better performance on the same test set (prediction accuracies: 84.5 and 85.2%, respectively). Entry 5 in **Table 7** displays models developed with Sun's training set, which is a big data set consisting of 3,024 agents (positives: 483; negatives: 2,541) with a threshold of 30 µM for defining hERG blockers and nonblockers. With the training set, Siramshetty et al. established two models by using LibSVM and RF methods, and their prediction accuracies on the test set were 71.0 and 74.0%, respectively. Our models offered much higher prediction accuracies (Conv-CapsNet: 83.3%; RBM-CapsNet: 86.3%). Entry 6 in **Table 7** shows models built with Siramshetty's training set T3 which were extracted from the ChEMBL database. In this training set, agents with a binding affinity of less than 1 µM were defined as hERG blockers, and those with a binding affinity of greater than 10 µM were defined as hERG nonblockers. With the training set, Siramshetty et al. established two models by using LibSVM and RF methods, and their prediction accuracies on their test set were 78.0 and 81.0%, respectively. Our Conv-CapsNet and RBM-CapsNet models gave prediction accuracies of 87.1 and 87.8%, respectively, which are obviously higher than those of LibSVM and RF models. Very recently, Cai et al. developed a deep learning model, termed deephERG, to predict hERG blockers with a large dataset containing 7,889 compounds (Cai et al., 2019). To make a comparison, we also used the same datasets to train and test hERG blocker prediction models. With the same validation set and evaluation method as those in Cai's work, our Conv-CapsNet (AUC = 0.974) and RBM-CapsNet (AUC = 0.978) showed a better performance than their deephERG (AUC = 0.967) (see **Table S5**). Collectively, for different training sets given here, the models developed by Conv-CapsNet and RBM-CapsNet were among the best models established by various machine learning methods.

## Discussion

Since the first capsule networks were proposed by Hinton's group in 2017 (Sabour et al., 2017), they have attracted considerable attention because of their performance. For example, despite the simple three-layer architecture of the original capsule networks, they have achieved state-of-the-art results with 0.25% test error on Mixed National Institute of Standards and Technology database (MNIST) without data augmentation, which is better than the previous baseline of 0.39% (Sabour et al., 2017). The excellent performance of capsule networks is mainly due to the introduction of the capsules and dynamic routing algorithms. A capsule is a set of neurons that forms a vector. These vectors contain information including the magnitude/prevalence, spatial orientation, and other attributes of the extracted feature. In the capsule networks, capsules are “routed” to any capsule in the next layer *via* a dynamic routing algorithm, which takes into account the agreement between these capsule vectors, thus forming meaningful part-to-whole relationships not found in standard CNNs. In other words, capsule networks are capable of catching and holding more fine information than traditional deep neuron networks, one benefit of which is that the amount of input data can be significantly reduced.

Although CapsNets were just proposed very recently, they have already been successfully applied in many fields (Afshar et al., 2018; Kumar, 2018; Lalonde and Bagci, 2018; Li et al., 2018; Liu et al., 2018; Mobiny and Van Nguyen, 2018; Qiao et al., 2018; Zhao et al., 2018; Peng et al., 2019). Among these applications, majorities are related to image recognition. For example, Afshar et al. (2018) established a CapsNet for brain tumor classification by recognizing brain magnetic resonance imaging (MRI) images and proved that it could successfully overcome the defects of CNNs. Kumar (2018) proposed a novel method for traffic sign detection using a CapsNet that achieved outstanding performance, the input of which was traffic sign images. Li et al. (2018) built a CapsNet to recognize rice composites from unmanned aerial vehicle (UAV) images. This is understandable because CapsNets were originally developed to overcome the defects associated with image recognition in the traditional deep learning networks.

In image recognition, the input data is a two-dimensional array. In this two-dimensional array data, adjacent data points are often highly correlated. Small changes in any points generally do not affect image recognition in traditional deep learning methods. However, in issues related to drug discovery, such as the evaluation of ADMET properties (like the prediction of hERG blockers), one-dimensional vectors that describe small molecular structures and properties are usually used as the network input, for example, molecular fingerprints and descriptors. Generally, there is no direct logical relationship between the components in each vector for this kind of input. Importantly small changes in vector components might have a significant impact on the entire molecular structure and its associated properties. Nevertheless, these small changes in vector components are often overlooked in traditional deep learning methods. In addition, the relative positions of vector components are often critical though there is no direct logical relationship between them because a vector component represents a substructure or property. In this situation, capsule networks, which adopt vector neurons, are expected to have a better performance in handling this kind of issue (like the hERG blocker modeling) than other deep scalar neuron networks.

As expected, the two established capsule networks, Conv-CapsNet and RBM-CapsNet, showed excellent performance in the classification of hERG blockade. Although this is the first application of capsule networks in the classification of hERG blockers/nonblockers, the established models are still among the best classification models for hERG blockers/nonblockers. There can be no doubt that the use of capsules or vector neurons is one of the main reasons that contribute to the excellent performance of our models. Here each capsule represents a combination of substructures and/or properties. Analogy to the case in image recognition, the length of each capsule is the probability that the combination of substructures or properties exists in a molecule, and the orientation may represent the relative position of the combination of substructures in a compound. Obviously, our capsule networks can learn some combinations of substructures and/or properties that are important for the hERG blockers or nonblockers. Even so, we have to acknowledge that the prediction models of hERG blockers/nonblockers developed by the new capsule networks are still like a black box. Some questions regarding the models are difficult to answer. For example, we can't exactly know what the combination of substructures and/or properties is, and which features are important to the model and which samples are hard to classify. Overall, the application of capsule networks in drug discovery is still in its infancy. Further improvement of capsule networks and applications in drug discovery are necessary in future studies.

## Data Availability Statement

The datasets generated for this study are available on request to the corresponding author.

## Author Contributions

SY designed the study. LH designed the algorithms. YwW and SJ executed the experiment and performed the data analysis. YwW mainly wrote the manuscript. SY, JZ, YfW and HF revised the manuscript. All authors discussed and commented on the manuscript.

## Funding

This work was supported by the National Natural Science Foundation of China (61876034, 81573349, 81773633, 21772130, and 81930125), National Science and Technology Major Project (2018ZX09711003-003-006; 2018ZX09711002-014-002, 2018ZX09201018, 2019ZX09301-135 and 2018ZX09711002-011-019), and 1.3.5 Project for Disciplines of Excellence, West China Hospital, Sichuan University.

## Conflict of Interest

The authors declare that the research was conducted in the absence of any commercial or financial relationships that could be construed as a potential conflict of interest.

## Supplementary Material

The Supplementary Material for this article can be found online at: https://www.frontiersin.org/articles/10.3389/fphar.2019.01631/full#supplementary-material
