# Supplementary material for: Capsule Networks Showed Excellent Performance in the Classification of hERG Blockers/Nonblockers
Source: Front Pharmacol. 2020 Jan 28;10:1631. doi: 10.3389/fphar.2019.01631 (PMC6997788; doi:10.3389/fphar.2019.01631)
Supplement: Supplementary file 1 [file DataSheet_1.docx]

Supplementary Material

# Supplementary Methods

## Deep belief networks (DBN)

DBN is generative neural network model with many layers of hidden explanatory factors, along with a greedy layer-wise unsupervised learning algorithm. They derived a fast, greedy algorithm to address a problem that learning was difficult in densely connected, directed belief nets. DBN is divided into two stages: pre-training and fine-tuning, and this learning approach makes it easy to interpret massive parameters in the deep hidden layers. The pre-training process of DBN can be achieved layer-by-layer from low to high layer to train these multilayer restricted Boltzmann machines (RBMs). In fine-tuning stage, the same way as traditional deeply feedforward neural network is used, and the parameters of RBMs from pre-training act as original data. The whole network is fine-tuned by back propagation (BP) with end-to-end.

## Convolutional neural networks (CNN)

CNN combines three architectural ideas to ensure some degree of shift and distortion invariance local receptive fields, shared weights or weight replication and sometimes spatial or temporal subsampling. Convolutional process is the biologically inspired variant of multilayer perceptions (MLPs), which exploits the spatially local correlation by enforcing a local connectivity pattern. The classical convolutional network is composed of alternating layers of convolution and pooling (i.e. subsampling). The aim of the first convolutional layer is to extract patterns found within local regions of the input images that are common throughout the dataset.

In CNN, convolution layer is regarded as features extraction layer and each feature map is a mapping plane in feature map layer. The fully connected layers aggregate the local information learned in the convolutional layers to do class discrimination and fully-connected network like DNNs (deep neuron networks).

## Multilayer perceptions (MLP)

MLP is one of the most common and popular neural architectures. It is widely used in many different areas like handwriting recognition, speech recognition, and time series prediction for instance. MLP consists of Perceptron-type neurons as processing units grouped together in layers. The computation in an MLP is feed forward only. The neurons processing the input to the net are grouped in the input layer. The output of the net is taken from the output neurons grouped in the output layer. Usually, there are also one or more layers between input and output layer called hidden layers, since they are not visible from the outside. Input neurons are just for making the data available to the net, they do not perform a computation. Any other single neuron computes as its so-called propagation function a weighted sum of its received inputs. Thus, the association of the weights and the inputs is linear.

# Supplementary Parameters

The contribution of each capsule *i* in PrimaryCaps to each capsule *j* in CapsNets is computed as follows:

$\hat{u}_{j|i}=W_{ij}\cdot u_{i}$ (1)

Parameter *u_i_* is considered as the output of capsule *i*., and $\hat{u}_{j|i}$ is a prediction vector representing a weighted contribution of each low-level capsule *i* to high-level capsule *j*. *W_ij_* is a weighting matrix that needs to be learned in backward pass calculations. Based on the degree of conformation between the capsules in the layer bellow and the parent capsules, coupling coefficients *c_ij_* are calculated using the following softmax function:

$c_{ij}=\frac{\exp(b_{ij})}{\sum_{k} \exp(b_{ik})}$ (2-1)

where *b_ij_* represents the log probability of the coupling strength between capsule *i* and capsule *j*. The value of *b_ij_* is initiated to 0 before the routing-by-agreement process. The value of *c_ij_* is then updated by an iterative dynamic routing process. If the coupling coefficient *c_ij_* between capsule *i* and capsule *j* is 1, the coupling coefficient between capsule *i* and other high-level capsules is 0.

To determine the total input vector *s_j_* of each high-level capsule *j*, a weighted sum over all prediction vectors is defined as follows:

$s_{j}=\sum_{i} c_{ij}\hat{u}_{j|i}$ (2-2)

The output vector *v_j_* of high-level capsule *j* is then computed using a non-linear squashing function:

$v_{j}=\frac{\left\| s_{j} \right\|^{2}}{1+\left\| s_{j} \right\|^{2}}\frac{s_{j}}{\left\| s_{j} \right\|}$ (2-3)

The loss *L_k_* for each capsule *k* in CapsNets is computed as follows:

$L_{k}=T_{k}\max(0,m^{+}-\left\| v_{k} \right\|)^{2}+\lambda(1-T_{k})\max(0,\left\| v_{k} \right\|-m^{-})^{2}$ (3)

where *T_k_*=1 if the predicted value is equal to the real, value and *T_k_*=0 otherwise. m^+^, m^-^, and λ are hyper-parameters that must be predefined. We used m^+^=0.9, m^-^=0.1, and λ=0.5, which have been shown to ensure the stability of the training process.

The RBMs used the following energy function as the loss function:

$E(v,h)=-(a^{T}\cdot v+b^{T}\cdot h+v^{T}\cdot\omega\cdot h)$ (4)

Where $v\in\left\{ 0,1 \right\}^{n}$ of visible layers is used to input data and $h\in\left\{ 0,1 \right\}^{n}$of hidden layers represent feature encoding for information of data. Parameters *a*, *b* and *w* represent weighting matrix.

# Supplementary Tables

**Table S1.** Hyperparameter settings of DBN.

| **Hyperparameter** | **Setting** |
| --- | --- |
| Numbers of RBM | 2 |
| Number of nodes in the Hidden feature layer | 256 |
| Number of nodes in the PrimaryCaps layer | 128 |
| Iteration of RBM | 100 |
| Iteration of network | 200 |
| Learning rate of RBM | 0.001 |
| Learning rate of network | 0.005 |
| Activation | Relu |
| Batch size | 148 |
| Optimizer | Adam |

**Table S2.**  Hyperparameter settings of CNN.

| **Hyperparameter** | **Setting** |
| --- | --- |
| Numbers of Convolutional layer | 3 |
| Numbers of Fully connected layer | 2 |
| Kernel_size | 3 |
| Filter | 16/32/64 |
| Number of nodes in the Hidden layer | 1024/2 |
| Learning rate of network | 0.001 |
| Activation | Relu |
| Batch size | 148 |
| Optimizer | Adam |

**Table S3.**  Hyperparameter settings of MLP.

| **Hyperparameter** | **Setting** |
| --- | --- |
| Numbers of Fully connected layer | 2 |
| Number of nodes in the Hidden layer | 256/2 |
| Learning rate of network | 0.001 |
| Activation | Relu/Sotfmax |
| Batch size | 148 |
| Optimizer | Adam |

**Table S4.** Hyperparameter search space considered for four standard machine learning baselines and optimized hyperparameter values in the best models.

| **Models** | **Hyperparameters** | **Search Space** | **Optimum Values** |
| --- | --- | --- | --- |
| SVM | *C* | [0.1, 0.2, 0.3, 0.4, 0.5, 0.6  0.7, 0.8, 0.9, 1.0] | 0.9 |
| kNN | Number of neighbors | [2, 5, 10, 50, 100] | 2 |
|  | *p* | [1, 2] | 2 |
| Logistic regression | *C* | [0.1, 0.2, 0.3, 0.4, 0.5, 0.6  0.7, 0.8, 0.9, 1.0] | 0.1 |
|  | intercept_scaling | [1, 2, 3] | 2 |
|  | max_iter | [100, 200, 300, 400, 500] | 100 |
| LightGBM | max_depth | [3, 5, 8, 10] | 5 |
|  | num_leaves | [21, 31, 41, 51] | 31 |
|  | learning rate | [0.01, 0.05, 0.1, 0.2, 0.3] | 0.2 |
|  | n_estimators | [10, 50, 100, 200] | 50 |
|  | min_data_in_leaf | [200, 400, 600] | 200 |
|  | min_gain_to_split | [10, 20, 30, 40] | 10 |
|  | feature_fraction | [0.5, 0.6, 0.7, 0.8, 0.9] | 0.8 |
|  | bagging_freq | [0, 1, 5, 10] | 0 |
|  | bagging_fraction | [0.6, 0.7, 0.8, 0.9, 1.0] | 0.8 |

**Table S5.** The area under the receiver operating characteristic curve (AUC) values of Conv-CapsNet, RBM-CapsNet and deephERG models across different decoy thresholds on Cai’s validation set.

| **Approach** | **10μM** | **20μM** | **40μM** | **60μM** | **80μM** | **100μM** |
| --- | --- | --- | --- | --- | --- | --- |
| Conv-CapsNet | 0.961 | 0.977 | 0.970 | 0.964 | 0.974 | 0.978 |
| RBM-CapsNet | 0.980 | 0.963 | 0.981 | 0.975 | 0.978 | 0.970 |
| deephERG | 0.883 | 0.899 | 0.950 | 0.962 | 0.967 | 0.958 |
